# A prospective cross-sectional study on quality of life and treatment satisfaction in type 2 diabetic patients with retinopathy without other major late diabetic complications

**DOI:** 10.1186/s12955-014-0131-2

**Published:** 2014-08-20

**Authors:** Nuria Alcubierre, Esther Rubinat, Alicia Traveset, Montserrat Martinez-Alonso, Marta Hernandez, Carmen Jurjo, Didac Mauricio

**Affiliations:** Institut de Recerca Biomèdica de Lleida, University of Lleida, Lleida, Spain; Department of Endocrinology and Nutrition, University Hospital Arnau de Vilanova, Lleida, Spain; Department of Ophthalmology, University Hospital Arnau de Vilanova, Lleida, Spain; Biostatistics Unit, Institut de Recerca Biomèdica de Lleida, University of Lleida, Lleida, Spain; Department of Endocrinology and Nutrition, Hospital Germans Trias Pujol, Carretera Canyet, S/N, 08916 Badalona, Spain

**Keywords:** Diabetic retinopathy, Quality of life, Treatment satisfaction, Specific questionnaires, Type 2 diabetes mellitus

## Abstract

**Background:**

To assess quality of life and treatment satisfaction in patients with type 2 diabetes mellitus with diabetic retinopathy (DR) using validated instruments, with comparison to patients without DR.

**Methods:**

A prospective cross-sectional study was designed to assess the influence of retinopathy on quality of life and treatment satisfaction in patients with type 2 diabetes mellitus who do not have any other advanced late complications that could interfere with these outcomes. We included 148 patients with DR and 149 without DR, all without other advanced diabetic complications. Quality of life was assessed using the Audit of Diabetes Dependent Quality of Life (ADDQoL) questionnaire, and treatment satisfaction was assessed using the Diabetes Treatment Satisfaction Questionnaire (DTSQ). Clinical and treatment variables related to diabetes were also collected. The degree of DR was classified according to the International Clinical Classification System. Multivariate linear regression models were used to model the ADDQoL and DTSQ scores according to sociodemographical and clinical characteristics, and to model the adjusted relationship of DTSQ with ADDQoL. In DR patients, a subanalysis assessed the relationship of these scores with the degree of retinopathy, severity of macular edema, and previous photocoagulation treatment.

**Results:**

DR was associated with significantly lower quality of life (p < 0.001), when examining the two general quality of life items and most of the specific domains. Concerning DTSQ, no difference was found in the total score, and only two domains that assess the perception of glycemic control (hyper- and hypoglycemia) showed a worse score in DR (p < 0.001 and p = 0.008, respectively). Quality of life was significantly affected by the severity of DR, and treatment satisfaction was significantly affected by the severity of macular edema. In the multivariate analysis, a significant effect of the interaction between diabetes duration, insulin therapy, and the presence of DR was found for both, ADDQoL and DTSQ.

**Conclusion:**

In the absence of other major complications, DR has a negative impact on quality of life in patients with type 2 diabetes. Further, treatment satisfaction was not affected by the presence of DR.

## Background

Diabetic retinopathy (DR) is a diabetes-specific ophthalmic complication that is still very common and often severe. It is the leading cause of preventable blindness in working-age adults [1,2]. In Spain, 15.6% of patients with type 2 diabetes mellitus are affected by the disease in its different stages, with a 4% prevalence of proliferative DR [3,4].

Improving quality of life is a primary goal when treating diabetic patients with DR. Diabetic visual impairment places the individual in a situation that can profoundly affect their quality of life [5–7]. Photocoagulation, the first-line and most frequently used treatment for diabetic retinopathy, has an adverse effect on health-related quality of life and treatment satisfaction in these patients [8].

We understand treatment satisfaction to be the “confirmation of expectations” for a patient, that is, the agreement between what the patients expects from the treatment and the results obtained [9]. Although new treatment standards advise reconciling traditional measures of vision assessment with the use of patient-reported outcome measures, choosing the most appropriate instrument to measure quality of life can be difficult [10]. The primary reason for using specific measures of diabetes-related quality of life is to provide an accurate, comprehensive, and personal assessment of this complication, analyzing its role in the patient’s life and maximizing the variability in the responses of patients with the same pathology or in certain groups [11].

In a recent review, Fenwick et al. established that DR is a threat to the quality of life of patients with type 1 or type 2 diabetes, especially in later stages, and illustrated the way in which different psychometric properties of the most frequently used scales can lead to very different outcomes [12]. However, the presence of other diabetic late complications is the very frequent in participants included in studies assessing quality of life in diabetic retinopathy [13,14]. For instance, in the study by Davidov et al., complications like coronary heart disease, nephropathy, peripheral vascular disease or cerebrovascular disease have been shown to be frequent in patients with retinopathy; in that study, patients had a mean of 2.4 associated co-morbidities and only 11% of them were free of any of this major health conditions [13]. In a very large study, the Andhra Pradesh Eye Disease Study [15], the authors showed the specific adverse impact in terms of quality of life of different eye diseases i.e. cataract, corneal diseases, retinal diseases, glaucoma and uncorrected refractive deffects. Specifically, in that study diseases of the retina with a very similar impact on visual health, like macular degeneration, showed an unfavourable impact on quality of life. Therefore, the specific impact of retinopathy on quality of life in type 2 diabetes deserves further research.

Few studies have assessed the impact of DR on the quality of life of type 2 diabetic patients. In addition, none of the instruments used in those studies, such as generic health status questionnaires or visual function scales, although they analyze patient-reported outcomes, can be considered sufficiently accurate to assess quality of life outcomes in diabetic patients [10,16]. Only one study examined satisfaction with photocoagulation treatment in patients with diabetic maculopathy or proliferative retinopathy, using the Diabetes Treatment Satisfaction Questionnaire (DTSQ), and it reported high levels of satisfaction despite not seeing improvements in visual acuity [17]. However, the results described in the scientific literature regarding the specific impact of DR in patients with type 2 diabetes mellitus should be treated with caution. The coexistence of other advanced diabetic complications, the small sizes of the samples, the heterogeneous distribution of the variables, and the joint analysis of results for type 1 and 2 diabetic patients all limit the utility of previous studies [10,13,15–19]. We have found no studies in the literature that investigate the specific impact of retinopathy on quality of life and treatment satisfaction in type 2 diabetic patients without other advanced diabetic complications using instruments specifically designed to assess these outcomes in diabetic patients. Thus, we think that further research is needed to explain the impact of retinopathy on these measures, specifically in type 2 diabetic patients in the absence of other associated complications.

This study hypothesizes that patients with DR have poorer self-perceived health-related quality of life and a lower degree of treatment satisfaction, regardless of the contribution of other late complications of diabetes (i.e., diabetic foot disease, cardiovascular disease, and diabetic nephropathy). We therefore present the first study, to our knowledge, specifically designed to assess the impact of DR in terms of quality of life and treatment satisfaction assessments in type 2 diabetic patients without other complications that might have confounding effects during the assessment. In addition, as a secondary objective, we set out to analyze the clinical and sociodemographic variables that may be associated with both assessments.

## Methods

A prospective, observational, cross-sectional study was designed. Patients were identified from a unpublished prospective study on cerebral microcirculation conducted by our research team in patients with and without retinopathy. From a total sample of 314 patients and based on the sample size calculation (see below), a total of a 299 were offered participation in the current study. All initially contacted patients fulfilled the predefined inclusion criteria and accepted the participation in the study; however, finally 2 subjects did not show up for the study visit even after additional efforts were made to include them. It should be noted that the study visits were scheduled independently of the retinopathy status at the best patient convenience to allow maximum participation. The group with DR (n = 148) consisted of patients between 40 and 75 years of age with type 2 diabetes mellitus who did not have macrovascular complications, a history of diabetic foot disease, macroalbuminuria, or renal failure. The group without DR (n = 149) consisted of patients in the same age range with type 2 diabetes mellitus without DR who also did not have the previously mentioned complications. The group of patients with DR included those who had any degree of DR, with or without microalbuminuria (albumin/creatinine ratio up to 300 mcg/g), but without macroalbuminuria or a history of diabetic foot disease. The group without retinopathy included participants without evidence of retinopathy on ophthalmologic evaluation. All patients had normal renal function (glomerular filtration rate > 60 ml/min). None of the study groups included patients with cardiovascular diabetic complications, which was confirmed by anamnesis and a review of patients’ medical history to verify the lack of heart failure, cerebrovascular disease, ischemic heart disease, or peripheral arterial disease. The recruitment of all the patients was primarily performed through the Department of Ophthalmology of the hospital and the enrollment of the patients was intended to include a similar number of subjects in terms of age and sex groups. This department is the main public referral center for our health care district and is responsible for conducting the screening, assessment and treatment of all DR cases. The study was discussed with all patients, and their written informed consent was obtained. The study was approved by the Ethics Committee of Hospital Arnau de Vilanova.

### Clinical variables

Clinical variables that were obtained are shown in Table 1. These variables were chosen for their relevance in relation to diabetes and/or for their potential impact on quality of life and treatment satisfaction. Laboratory tests were performed on blood and urine samples that were collected after a 12-hour fast using standard laboratory methods. Blood pressure was measured in the sitting position after resting for 10 minutes. Medical records were reviewed to rule out any known cardiovascular events. Hypertension or dyslipidemia was considered present when the patient was being treated with antihypertensive or lipid-lowering drugs, respectively. The use of antiplatelet agents and psychotropic drugs was also recorded. None of the patients had renal failure, defined as a glomerular filtration rate < 60 ml/min according to the Modification of Diet in Renal Disease formula. The glycosylated hemoglobin concentration is expressed in National Glycohemoglobin Standardization Program/Diabetes Control and Complications Trial units. To determine the level of physical activity, the concept of active leisure time that was developed by Bernstein et al. was used, which defines a sedentary person as one who spends less than 10% of his/her daily energy expenditure performing any physical activity that requires at least 4 METs (The Metabolic Equivalent) (equal or greater physical activity expenditure than brisk walking for 30 minutes) [20]. MET is the ratio of a person’s working metabolic rate relative to his/her resting metabolic rate and is equivalent to a caloric consumption of 1 kcal/kg/hour [21]. Visual acuity was measured using the Snellen chart. For statistical analysis, Snellen acuities were converted to equivalent values using logarithm of the minimum angle of resolution [22].

**Table 1:** **Demographic and clinical characteristics of the study groups**

**Characteristics**	**No retinopathy (n = 149)**	**Retinopathy (n = 148)**	**p-value**
Sex (men)	78 (52.3%)	73 (49.3%)	0.685
Age (years)	57.9 (19.26)	60.5 (8.77)	0.042
Education			<0.001
Not even primary	13 (8.7%)	25 (16.9%)	
Complete primary	79 (53.1%)	90 (60.8%)	
Secondary high cycle	39 (26.1%)	30 (20.3%)	
Graduate or higher	18 (12.1%)	3 (2.0%)	
Ethnicity			0.990
Non caucasian	5 (3.3%)	6 (4.0%)	
Smoking			0.483
Yes	31 (21.1%)	31 (21.1%)	
No	65 (44.2%)	74 (50.3%)	
Former smoker	51 (34.7%)	42 (28.6%)	
Diabetes duration (years)	6.0 [3,10]	11.0 [7.2,9.1]	<0.001
HbA1c (%)	7.1 [6.5,7.9]	8.1 [7.2,9.1]	<0.001
Hypertension	74 (49.7%)	94 (63.5%)	0.022
Dyslipidemia	65(43.6%)	66 (44.6%)	0.959
Antiplatelet agents	46(30.9%)	68 (45.9%)	0.011
Psychotropic drugs	35 (23.5%)	48 (32.4%)	0.112
Serum creatinine (mg/dl)	0.80 (0.2)	0.81 (0.2)	0.830
Systolic blood pressure (mmHg)	134.4 (15.5)	144.4 (20.1)	<0.001
Diastolic blood pressure (mmHg)	76.5 (10.4)	77.1 (11.0)	0.634
Waist (cms)	104.1 (12.1)	107.26 (11.3)	0.010
BMI (kg/m^2^)	31.25 (5.1)	31.92 (5.5)	0.240
Diabetes treatment			<0.001
OAD	96 (64.4%)	65 (43.9%)	
OAD + insulin	13 (8.7%)	62 (41.9%)	
Insulin	4 (2.7%)	18 (12.2%)	
Diet	36 (24.2%)	3 (2.0%)	
Visual acuity			<0.001
< = 0.2	3 (2.0%)	30 (20.4%)	
0.2-0.4	11 (7.4%)	8 (5.4%)	
0.4-0.6	17 (11.4%)	24 (16.3%)	
0.6-0.8	44 (29.5%)	36 (24.5%)	
>0.8	74 (49.7%)	49 (33.4%)	
Physical activity			0.304
More than 25 minutes/day	87 (58.4%)	96 (64.9%)	
Less than 25 minutes/day	62 (41.6%)	52 (35.1%)	

Values are shown as mean ± SD or median ± interquartile range for age, diabetes duration, HbA1c, systolic blood pressure, diastolic blood pressure, waist and BMI; frequency (%) for all other variables. HbA1c: glycated haemoglobin; BMI: body mass index; OAD: oral antidiabetic agents. The p-values correspond to the unadjusted univariate analysis that compares the difference for each variable between patients with and without retinopathy.

All the questionnaires were administered individually by personal interview by a single trained interviewer (N.A.), after the diagnostic assessment at the Department of Ophthalmology. The response rate was 99.3%.

### Quality of life

At the time of study design in 2009, an instrument to measure quality of life in diabetic patients that was properly validated for Spain was sought. The Audit of Diabetes-Dependent Quality of Life (ADDQoL-19) was specifically designed to measure the individual’s perspective regarding the impact of diabetes and its treatment on quality of life [23]. The first two items are general in nature and are scored separately: the first measures current quality of life, scoring from −3 (extremely poor) to +3 (excellent), and the second assesses the overall impact of diabetes on quality of life, scoring from −3 (maximum negative impact of diabetes) to +1 (maximum positive impact of diabetes). Individual items ask about 19 specific areas of life, as indicated in Table 2. Each question is rated on a 5-point scale (−3 to 1), which is later weighted by the importance attributed to the particular dimension by the patient (0–3). In addition, five of the items that may not be relevant for some people have a preliminary question that determines the relevance of the dimension, and it is ignored if it is not applicable. A final score is then obtained that ranges from −9 (maximum negative impact) to +3 (maximum positive impact) for each dimension. This questionnaire allows for the calculation of a final weighted score of the effects of diabetes and its treatment on the quality of life of patients—the average weighted impact—ranging from −9 (maximum negative impact of diabetes) to +3 (maximum positive impact of diabetes) [24]. The ADDQoL has been validated in studies of Spanish patients with type 2 diabetes [25,26].

**Table 2:** **Summary of Audit of Diabetes Dependent Quality of Life (ADDQoL) measures**

**Life domains**	No retinopathy*	Retinopathy*	**p-value**
Present QoL	0.99 (1.00)	0.39 (1.19)	<0.001
	1 [0,2]	1 [ 0,1]	
Diabetes specific QoL	−0.50 (0.74)	−1.08 (1.00)	<0.001
	0 [−1,0]	−1[−2,0]	
Leisure	−0.42 (1.29)	−1.06 (2.01)	<0.001
	0 [0,0]	0 [−2,0]	
Work life	−0.38 (1.19)	−1.05 (2.02)	0.022
	0[0,0]	0 [0,0]	
Travels	−0.41 (1.33)	−0.79 (1.78)	0.009
	0 [0,0]	0 [0,0]	
Holidays	−0.29 (1.13)	−0.46 (1.56)	0.233
	0 [0,0]	0 [0,0]	
Physical ability	−0.63 (1.60)	−1.74 (2.54)	<0.001
	0 [0,0]	0 [−3,0]	
Family life	−0.34 (1.13)	−0.92 (2.18)	0.018
	0 [0,0]	0 [0,0]	
Friends/social life	−0.11 (0.51)	−0.47 (1.44)	0.010
	0 [0,0]	0 [0,0]	
Personal relationship	−0.18 (1.10)	−1.04 (2.31)	<0.001
	0 [0,0]	0 [0,0]	
Sex life	−0.64 (1.63)	−2.05 (2.60)	<0.001
	0 [0,0]	−1 [−4,0]	
Physical appearance	−0.16 (0.92)	−0.47 (1.58)	0.065
	0 [0,0]	0 [0,0]	
Self-confidence	−0.36 (1.25)	−0.92 (1.99)	0.006
	0 [0,0]	0 [0,0]	
Motivation	0.51 (1.65)	−1.33 (2.33)	<0.001
	0 [0,0]	0 [−2,0]	
Society/people’s reaction	−0.10 (0.53)	0.38 (1.40)	0.084
	0 [0,0]	0 [0,0]	
Future	−1.51 (2.59)	−2.53 (3.03)	<0.001
	0 [−2,0]	−2[−6,0]	
Finances	−0.13 (0.72)	−0.56 (1.84)	0.022
	0 [0,0]	0 [0,0]	
Living conditions	−0.08 (0.60)	−0.25 (1.03)	0.062
	0 [0,0]	0 [0,0]	
Dependence	−0.15 (0.66)	−0.96 (2.00)	<0.001
	0 [0,0]	0 [0,0]	
Freedom to eat	−3.00 (3.25)	−3.85 (3.78)	0.074
	−2 [−6,0]	−2.5 [−9,0]	
Freedom to drink	−1.48 (2.73)	−1.91 (3.04)	0.129
	0 [−1,0]	0[−2,0]	
Average weighted impact score	−0.58 (0.74)	−1.22 (1.17)	<0.001
	−0.35 [−0.78,-0.06]	−0.88 [−1.76,-0.38]	

*mean (standard deviation) in first line, and median [P25,P75] in second line. The p-values correspond to the unadjusted univariate analysis that compares the difference for each variable between patients with and without retinopathy.

### Treatment satisfaction

The Diabetes Treatment Satisfaction Questionnaire (DTSQ), designed to assess the degree of treatment satisfaction, was used [27]. This instrument has been validated for the Spanish population [28]. It consists of eight questions, two of which are scored separately (frequency of hyper- and hypoglycemia). All items have seven possible responses, and the score ranges between 0 and 6. Overall satisfaction is expressed with an overall score of 0 to 36, with higher values indicating higher treatment satisfaction. This questionnaire has been recommended by the World Health Organization and the International Diabetes Federation as a valid instrument that enables the accurate measurement of treatment satisfaction for type 1 or 2 diabetic patients [29].

### Assessment of diabetic retinopathy

DR and macular edema were classified according to the International Clinical Classification System [30]. This classification is widely used and determines the degree of retinopathy, including macular edema, by objective criteria. The classification categories are as follows: 1) no diabetic retinopathy, 2) mild non-proliferative diabetic retinopathy, 3) moderate non-proliferative diabetic retinopathy, 4) severe diabetic retinopathy, and 5) proliferative diabetic retinopathy. For the diagnosis of macular edema, the Early Treatment Diabetic Retinopathy Study Research Group criteria were used as reference, and the concept of macular edema was applied in those cases where it was clinically significant [31]. If the eyes were not equivalent, the participant was classified according to the eye with more severe stage.

### Sample size

To determine the sample size, an estimation of the standard deviation for the ADDQoL domains was obtained from previous studies [32]. We used the highest reported SD (value 3.37) and predefined a statistical power of 80% and a significance level of 5%. In order to detect a predefined mean difference of at least 1.2 in the ADDQoL score between the study groups and expecting no more than 15% of individuals being lost to follow-up, a minimum sample size of 146 per group was required.

### Statistical analysis

Comparative analyses between groups of diabetic patients with and without retinopathy were performed using the Mann–Whitney U test for quantitative variables and the chi-squared test or Fisher test, as appropriate, for qualitative variables. Graphical analyses of smoothed trends (using a span of 0.95) were performed to explore the possible interactions between the groups and treatment with insulin and diabetes duration, as well as the non-linear relationship of diabetes duration with the ADDQoL and DTSQ scores. Afterwards, two multivariate linear regression models were fit in order to assess whether retinopathy remained a significant contributor to the differences in ADDQoL and DTSQ scores after accounting for treatment with insulin, diabetes duration, and all other variables that had a significant contribution, according to the likelihood ratio test (LR test), to explain changes in each score. Each of the demographical and clinical variables were tested for contribution to the main outcome measures, starting from a model only with the DR identifier adding the variable with the most significant contribution (lower LR test p-value) to the previous model, and so on by refitting the significance of the contribution of each of the remaining variables to the model until no statistical contribution was obtained. A final multivariate linear regression model was fit to assess the association of the reported satisfaction with diabetes treatment and the perceived quality of life, using the same methodology. Additionally, a subanalysis in the group with retinopathy was performed to assess the association of the level of retinopathy, the severity of macular edema, and previous photocoagulation treatment with the ADDQoL and DTSQ scores using a Kruskal-Wallis test and a quantile regression model for median trend for the first two variables and the Mann–Whitney test for the photocoagulation treatment. A significance level of 0.05 was used. The analyses were performed using R statistical software.

## Results

The primary clinical and sociodemographic characteristics and their comparison between the two groups are shown in Table 1. Patients with retinopathy had a slightly higher average age because fewer patients with DR were identified between 40 and 50 years of age. Patients with DR had less schooling, a longer duration of diabetes, greater glycosylated hemoglobin levels, and greater frequency of arterial hypertension. As expected, patients with DR had higher urinary albumin concentrations and higher systolic blood pressure. Although body mass was not different, patients with retinopathy had a higher waist circumference. Patients with DR also received more complex treatment which is related to the longer duration of diabetes.

The distribution of each degree of retinopathy was as follows: 40.7% with mild nonproliferative DR, 35.9% with moderate nonproliferative DR, and 23.4% with severe proliferative DR. Diabetic macular edema was present in 52 patients (35.9% of those with DR). Of this percentage, edema was clinically significant in 21.4% and not clinically significant in 14.5%. For the DR group, a significant association between the degree of DR and the presence of clinically significant macular edema was observed (p < 0.0001). A significant association was also observed between the degree of DR and previous photocoagulation treatment (p < 0.0001).

### Health-related quality of life using ADDQoL scores

Scores representing diabetes-related quality of life were significantly lower in patients with DR in the following specific domains: leisure, work, freedom to travel, physical ability, family and social life, emotional and sexual relationships, self-confidence, personal finances, motivation, future, and dependence (Table 2). The average weighted impact score showed a median value of −0.35 (95% CI: 0.78-0.06) in the group without DR and −0.88 (95% CI: 1.76-0.38) in the group with DR (p < 0.001). This indicates a negative impact of diabetes on quality of life in both groups, with a greater negative impact in the group with DR. The first two items that assess the current quality of life and overall impact of diabetes on quality of life also had significantly lower outcomes in patients with DR (p < 0.001; Table 2).

In patients with DR, the observed median quality of life scores were −0.65, −0.94, and −1.03 for mild, moderate, and severe DR grades, respectively, and quality of life decreased significantly in relation to the degree of DR (the estimated trend for the median was −0.22 [−0.36, −0.03]), but not in relation to the severity of macular edema (estimated trend for the median of −0.09 [−0.23, 0.02]).

**Figure 1:**
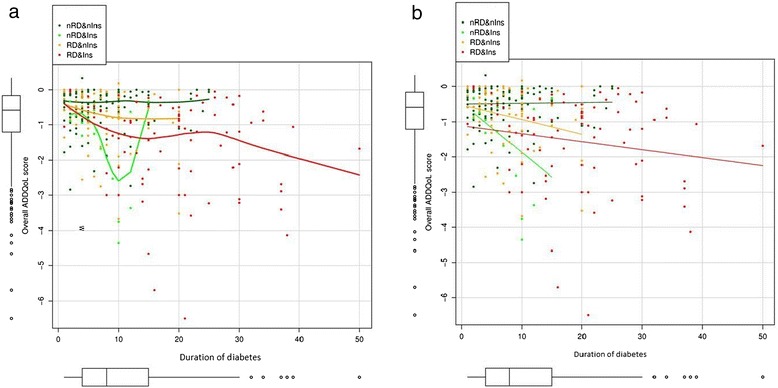
**Relationship between ADDQoL and duration of diabetes by groups defined by the presence of diabetic retinopathy (DR) or absence of retinopathy (nRD), and insulin treatment (Ins) or not (nIns).** Panel **a** shows the smoothed relationship while panel **b** shows the fitted trend assuming a linear relationship. **a**. Smoothed relationship between Audit Diabetes Dependent Quality of Life (ADDQoL) and duration of diabetes. **b**. Linear relationship between ADDQoL and duration of diabetes.

The multivariate analysis revealed a second-order interaction between diabetes duration, the presence of DR, and insulin therapy (p = 0.005, Table 3). Quality of life was significantly associated with three factors that interacted with each other: diabetes duration, treatment with insulin, and the presence or absence of DR (Figure 1a, electronic supplementary material). Therefore, the differences in quality of life attributable to retinopathy cannot be quantified without taking into account the impact of diabetes duration and insulin therapy. Besides, the association with diabetes duration is not linear and there is a general decrease in quality of life during the initial years of the disease that smoothes and reverts for patients with a long duration of diabetes, especially those with insulin therapy. Quality of life improved with increasing duration of the disease, especially in those patients without DR who were being treated with insulin, which consisted of 17 patients and was smaller than the other groups, consisting of 68, 80 and 132 patients (Figure 1b, electronic supplementary material). When quantitative variables in the multivariate model were kept constant, quality of life had an association with the duration of diabetes that depended on the presence or absence of DR or insulin therapy. Regarding the effect of the quantitative variables on quality of life, patients over 65 years rated their quality of life higher (p = 0.016) than younger patients. Waist circumference had an inverse relationship, and therefore a negative slope, with the assessment of quality of life (p = 0.010).

**Table 3:** **Multivariate linear regression for the audit of diabetes dependent quality of life**

**Coefficients**	**Estimate**	**Standard deviation**	**p-value**
Intercept	0.8020	0.5098	0.1168
Diabetes duration (years)	−0.0269	0.0418	0.5203
Diabetes duration-squared (years^2^)	0.0008	0.001	0.6305
Retinopathy	0.0108	0.3645	0.9762
Insulin	1.6923	0.8318	0.0428
Age >65	0.2798	0.1155	0.0161
Ethnicity	−1.0309	0.27292	0.0001
Waist (centimetres)	−0.0116	0.0044	0.0101
Diabetes duration* DR	−0.0483	0.0851	0.5705
Diabetes duration-squared * DR	0.0003	0.0039	0.9302
Diabetes duration * insulin	−0.7326	0.2457	0.0031
Diabetes duration-squared* insulin	0.0394	0.0152	0.0102
DR * insulin	−1.9684	0.9583	0.0409
Diabetes duration * DR * insulin	0.7348	0.2590	0.0049
Diabetes duration-squared* DR * Insulin	−0.0394	0.0156	0.0122

Multiple R-squared: 32.69%. *stands for the existence of interactions between variables. DR: diabetic retinopathy.

### Treatment satisfaction using DTSQ

The DTSQ median score was not different between patients with or without retinopathy (mean score: 26 vs 27, respectively; p = 0.236) (Table 4). However, differences were found in some of the specific questionnaire items. There was a greater perception of having suffered hyper- and hypoglycemic episodes in patients with DR, with a statistically significant difference (p < 0.001 and p = 0.008, respectively). These patients also expressed greater discomfort regarding treatment and its results (p < 0.001).

**Table 4:** **Summary of the Diabetes Treatment Satisfaction Questionaries (DTSQ) measures**

**DTSQ items**	**No retinopathy**	**Retinopathy**	**p-value**
Hyperglycemias frequency perception	2.46 (2.23)	3.58 (2.19)	<0.001
	2 [0,4]	0 [2,6]	
Hypoglycemias frequency perception	0.99 (1.63)	1.64 (2.12)	0.008
	0 [0,2]	0 [0,3]	
Current treatment	5.00 (1.48)	4.8 (1.53)	0.445
	5 [5,6]	5[4,6]	
Convenience	4.99 (1.63)	4.41 (1.82)	<0.001
	6 [5,6]	5 [4,6]	
Flexibility	3.34 (2.49)	2.91 (2.36)	0.057
	4 [0,6]	3 [0,5]	
Understanding	4.37 (1.89)	4.45 (1.85)	0.808
	5 [3,6]	5 [3,6]	
Recommend to others	3.19 (2.23)	3.00 (2.27)	0.362
	3 [1,5]	3 [0.75,5]	
Continue with	5.81 (0.85)	5.76 (1.00)	0.642
	6 [6,6]	6 [6,6]	
Final score	26.73 (5.61)	25.43 (6.70)	0.236
	27 [23,30]	26 [21.75,31]	

Mean (standard deviation) in first line, and median [P25,P75] in second line. The p-values correspond to the unadjusted univariate analysis that compares the difference for each variable between patients with and without retinopathy.

**Figure 2:**
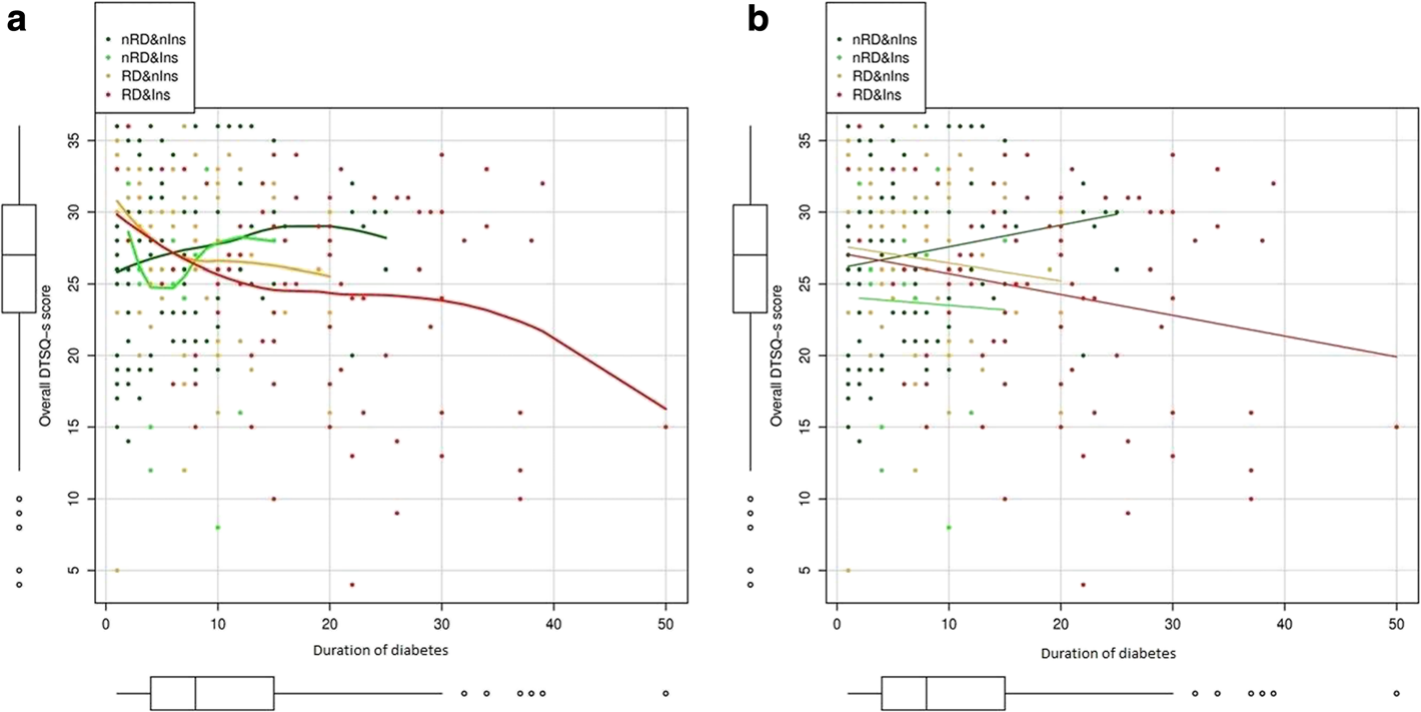
**Relationship between Diabetes Treatment Satisfaction Questionnaire status version (DTSQ-s) and duration of diabetes mellitus by groups defined by the presence of diabetic retinopathy (DR) or absence of diabetic retinopathy (nRD), and insulin treatment (Ins) or not (nIns).** Panel **a** shows the smoothed relationship while panel **b** shows the fitted trend assuming a linear relationship. **a**. Smoothed relationship between Diabetes Treatment Satisfaction Questionnaire status version and duration of diabetes in patiens with and without diabetic retinopathy. **b**. Linear relationship between Diabetes Treatment Satisfaction Questionnaire status version and duration of diabetes in patiens with and without diabetic retinopathy.

In the group of patients with DR, the subanalysis showed how treatment satisfaction was significantly affected by the severity of macular edema (medians of 28.0, 25.0, and 25.0; trend for the median −1.50 [−4.17, −0.08]). Multivariate analysis revealed that patients with DR had lower treatment satisfaction in relation to the duration of diabetes (p = 0.016, Table 5). This relationship was influenced by two factors: insulin therapy and the presence of DR. An approximately linear relationship was seen with diabetes duration, which differs significantly between patients with and without DR (Figure 2a, electronic supplementary material). In the group of patients without DR who were not receiving insulin therapy, treatment satisfaction increased as the duration of diabetes increased (Figure 2b, electronic supplementary material). Physically active patients had greater treatment satisfaction (p = 0.001), and former smokers had lower satisfaction compared with the group of smokers (p = 0.044) (Table 5).

**Table 5:** **Multivariate linear regression for the diabetes treatment satisfaction questionaries**

**Coefficients**	**Estimate**	**Standard deviation**	**p-value**
Intercept	24.7585	1.0030	< 2e −16
Insulin	−1.6874	0.9514	0.077
Diabetes duration (years)	0.1571	0.0907	0.084
Retinopathy	1.7524	1.1792	0.138
Physical activity > 20 minutes	2.3979	0.7176	0.001
Tobacco use:			
Smoker	0.6628	0.92503	0.474
Former smoker	−1.6248	0.8016	0.044
Diabetes duration * DR	−0.2587	0.1070	0.016

Multiple R-squared: 11.45%. *stands for the existence of interactions between variables. DR: diabetic retinopathy.

### Relationship between quality of life and treatment satisfaction

**Figure 3:**
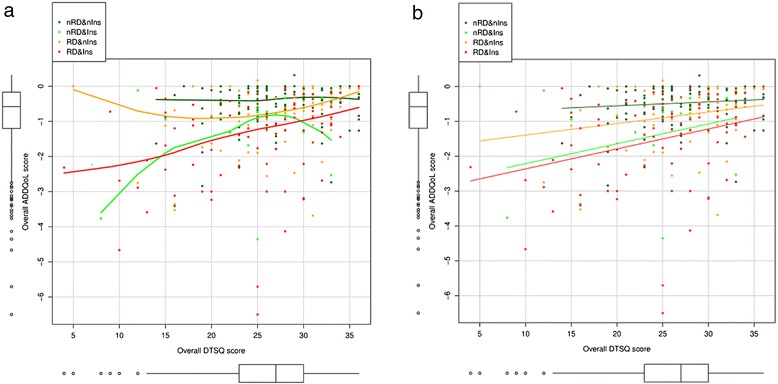
**Relationship between Audit Diabetes Dependent Quality of Life (ADDQoL) and Diabetes Treatment Satisfaction Questionnaire (DTSQ) by groups defined by the presence of diabetic retinopathy (DR) or absence of retinopathy (nDR), and insulin treatment (Ins) or not (nIns).** Panel **a** shows the smoothed relationship while panel **b** shows the fitted trend assuming a linear relationship. **a**. Smoothed relationship between Audit Diabetes Dependent Quality of Life and Diabetes Treatment Satisfaction Questionnaire. **b**. Linear relationship between Audit Diabetes Dependent Quality of Life and Diabetes Treatment Satisfaction Questionnaire.

The same interaction between DR, insulin therapy and diabetes duration was significant when adding treatment satisfaction for explaining the variability observed in the quality of life assessment (Table 6). Treatment satisfaction shows a significant relationship with quality of life that is dependent on insulin therapy but not on DR. Relationship between treatment satisfaction and the assessment of quality life was only significant in patients treated with insulin, regardless of whether they had DR or not (Figure 3a and b, electronic supplementary material).

**Table 6:** **Relationship between treatment satisfaction and quality of life**

**Coefficients**	**Estimate**	**Standard deviation**	**p-value**
Intercept	0.3086	0.5783	0.594
Treatment satisfaction (DTSQ) total score	0.0166	0.0111	0.137
Insulin	0.3869	0.9188	0.674
Retinopathy (DR)	−0.0256	0.3506	0.942
Diabetes duration (years)	−0.0343	0.0404	0.396
Diabetes duration-squared (years^2^)	0.0011	0.0017	0.528
Age >65	0.2809	0.1108	0.012
Waist	−0.0109	0.0043	0.012
Insulin * DR	−1.8622	0.9199	0.044
DR * Diabetes duration	−0.0424	0.0818	0.604
DR * Diabetes duration-squared	0.0002	0.0038	0.947
Insulin * Diabetes duration	−0.6268	0.2367	0.009
Insulin * Diabetes duration-squared	0.0330	0.0146	0.025
DR * Insulin * Diabetes duration	0.6405	0.2492	0.011
DR * Insulin * Diabetes duration-squared	−0.0333	0.0150	0.028
DTSQ total score * insulin	0.0433	0.0157	0.010

Multiple R-squared: 38.49%. *stands for the existence of interactions between variables. DTSQ: diabetes treatment satisfaction questionnire; DR: diabetic retinopathy.

## Discussion

We have shown that patients with type 2 diabetes with DR and no other advanced late complications report a lower quality of life. The inclusion of only patients without other complications that may have a significant clinical impact on quality of life is a particularly noteworthy element of this study. To our knowledge, this is the first study designed with this specific objective. The results revealed lower ADDQoL scores in the group of patients with DR. Quality of life was affected by the presence and degree of DR, insulin therapy and the duration of the disease. Although the DTSQ showed no statistically significant difference in the final score between study groups, the two specific items that assess perceived glycemic control showed significantly worse scores in patients with DR. Treatment satisfaction was affected over time by the presence of DR, insulin therapy and the severity of macular edema.

The results show the clearly negative impact of DR on quality of life in a large sample with enough statistical power that comprised only subjects with type 2 diabetes mellitus who did not have any other advanced diabetic complications that could exert important confounding or modifying effects on the main study outcome variables. Additionally, the assessment of quality of life and treatment satisfaction using instruments specifically designed and validated for this purpose strengthens these results [25,26,28].

The first approaches to health-related quality of life in the field of diabetes were made through the assessment of health status. However, it is important to note that even if health status is an area of health-related quality of life, there are other domains to consider (e.g., emotional well-being, personal care, physical, social, and cognitive functioning). Quality of life is a recent construct that is conceptually complex and composed of objective and subjective domains. Assessing only health status is unlikely to convey a precise picture of health-related quality of life [33]. With this approach, an optimal health outcome may not be considered an excellent result in terms of quality of life or perceived health status of the patient.

Tung et al. analyzed the impact of the different stages of DR in type 2 diabetic patients with associated nephropathy in Taiwan using utility values [18]. This approach allows the quantification of the level of impairment of the person and the level of functioning in everyday life that is affected by the disease. It allows for an assessment of quality of life by determining health status and is useful for obtaining quality-adjusted life years (QALYs). The results illustrated the influence of the severity of DR and age on utility values. Older age and different degrees of DR had a strong impact on the QALYs after adjusting for other possible non-ophthalmologic health-related factors. Such instruments have demonstrated their validity in cost-utility or economic evaluation studies; however, we do not consider an index that was developed to measure health status suitable for the assessment of quality of life. Assessing DR using a patient’s health status would explain the differences between this and our study regarding age. Furthermore, the presence of diabetic nephropathy in a significant proportion of patients, as in the study by Tung et al. [18], can exert a confounding effect on the results. In another study, the Los Angeles Latino Eye Study [16], a large sample of Hispanic patients with type 2 diabetes mellitus and a high burden of comorbidities was studied. The impact of DR and its severity on quality of life was studied using a generic health questionnaire and a vision-specific functioning and quality of life questionnaire designed for people with visual disabilities. The results of the study showed that patients with DR had lower scores on both the Medical Outcomes study 12-Item Short Form Health Survey and the National Eye Institute Visual Function Questionnaire [16]. This association was influenced by both the severity and the laterality of DR. However, the presence of other comorbidities may have had an additive effect on the outcome variables. Chung et al. explored the factors associated with quality of life in a large cohort of Korean patients with type 2 diabetes using the ADDQoL without cardiovascular disease or end-stage renal disease, although patients may have had peripheral neuropathy, retinopathy, and/or diabetic nephropathy [14]. They found that insulin therapy, depressive symptoms, and family history of diabetes were associated with lower quality of life, especially in younger patients. We also found lower ADDQoL scores in younger patients receiving insulin therapy. However, in contrast to our results, the Korean study did not show a significant impact of microvascular complications on quality of life.

Fenwick et al. investigated the impact of DR and macular edema on quality of life in type 1 and 2 diabetic patients using the EuroQoL-5D as a generic utility measure and demonstrated the inadequacy of the instrument and its low sensitivity for the assessment of vision-related quality of life [19]. Moreover, the same authors also assessed the impact of DR with the Vision and Quality of Life Index as a specific instrument for vision and established its validity for measuring the impact that this complication can have on the quality of life of type 1 and 2 diabetic patients. This index, however, did not appear sufficiently sensitive for assessing the impact of the severity of diabetic macular edema, the severity of DR, or the level of visual acuity [34]. However, the coexistence of other diabetic complications and the use of data from patients with either type 1 or type 2 diabetes does not allow for the comparison of these results with our results.

Our results also illustrate the impact of insulin therapy on quality of life and treatment satisfaction. We could not identify any studies specifically designed to assess the issue of treatment satisfaction in patients with type 2 diabetes and retinopathy. Mozaffarieh et al. observed an influence of age on treatment satisfaction in a mixed sample of type 1 and 2 diabetic patients with retinopathy who were treated with photocoagulation [17]. Younger patients had lower DTSQ scores. Using the same instrument, Redekop et al. investigated the clinical and sociodemographic characteristics associated with treatment satisfaction in a sample of Dutch patients with type 2 diabetes mellitus [35]. Lower levels of satisfaction were observed in univariate analyses of patients with diabetic complications, but this association was not maintained after adjustment for age, insulin, and glycated haemoglobin levels. In our study, treatment satisfaction was significantly affected by the patient’s perceived glycemic control, the duration of the disease, the degree of physical activity and smoking.

Regarding the limitations of this study, the study design inherently allows us to study only associations and not causality. Additionally, the fact that diabetes duration is a major factor in the development of DR caused a discrete bias in age distribution, with a lower proportion of younger patients in the retinopathy group. While other major complications that could affect quality of life and treatment satisfaction have been ruled out, potential symptoms of peripheral neuropathy that might influence these aspects were not assessed. However, none of the patients had advanced neuropathy leading to serious complications such as diabetic foot disease. The representativeness of the study groups is partially limited and the conclusions may not be generalized to all the population of patients with type 2 diabetes. The results may be applicable to patients with or without retinopathy in the absence of other advanced late diabetic complications that is an important proportion of patients in the Spanish population according to recent reports [36].

In conclusion, in type 2 diabetic patients, the presence of DR is associated with poorer quality of life. Although satisfaction with the overall treatment is not different between the two groups of patients, it is influenced by other clinical variables. The results of this study are relevant because they demonstrate for the first time the negative influence of DR, regardless of the presence of other complications, on the quality of life of type 2 diabetic patients in a study specifically designed for that purpose. Clinicians should be aware that quality of life is one of the primary objectives of diabetes treatment. In addition, these findings should be taken into account in clinical practice when treating patients with DR and type 2 diabetes mellitus. Apart from the benefits in terms of visual outcomes, early identification and treatment of patients with DR would have a positive impact on the different dimensions of the patient’s quality of life. However, the potential impact of the early diagnosis and treatment of DR on quality of life deserves the performance of specific intervention studies to address this issue. We also believe that there is a need for additional studies to conduct a linguistic and psychometric validation of new measures of quality of life and treatment satisfaction and to develop measurement tools that would allow the assessment of the impact of DR treatments on the patient.
